# Unmasking Hereditary Fructose Intolerance: Turning a Rare Diagnosis Into a Path for Healing

**DOI:** 10.1002/ccr3.71654

**Published:** 2025-12-15

**Authors:** Rajat Kumar Shah, Sajjad Ahmed Khan, Bijita Aryal, Aakash Khatiwada, Binita Gurubacharya, Shamin Parajuli

**Affiliations:** ^1^ Birat Medical College Teaching Hospital Morang Nepal; ^2^ International Friendship Children Hospital Kathmandu Nepal; ^3^ Chirayu National Hospital and Medical Institute Kathmandu Nepal

**Keywords:** fructose, gastroenterology, hereditary fructose intolerance, multidisciplinary care, pediatrics, sorbitol (FSS)‐free diet, sucrose

## Abstract

Early diagnosis of children with hereditary fructose intolerance, which can be achieved by proper history taking, thorough clinical examination, response to diet, and histopathological examination, followed by effective management with a lifelong fructose, sucrose, and sorbitol‐free diet, ensures good prognosis and normal life.

## 1 Introduction

Hereditary fructose intolerance (HFI) is a rare autosomal recessive metabolic disorder caused by a mutation in the ALDOB gene which encodes for the enzyme aldolase B [1]. The prevalence of HFI ranges from 1 in 20,000 to 1 in 60,000 with no sex prediction [2]. After fructose intake, it is phosphorylated to fructose 1‐phosphate (F 1‐P) which is then catabolized by aldolase B into glyceraldehyde and dihydroxyacetone phosphate. In the absence of this enzyme, F 1‐P accumulates, which is toxic to the liver and inhibits gluconeogenesis and glycogenolysis [2, 3].

Dietary exposure to fructose, sucrose, or sorbitol in HFI is characterized by metabolic disturbances like hypoglycemia, lactic academia, hypophosphatemia, hyperuricemia, and hypermagnesemia, with clinical manifestations of nausea, vomiting, abdominal distress, and failure to thrive [4]. In patients with HFI, prolonged consumption of a high fructose diet may lead to liver damage leading to its enlargement and steatosis as well as non‐alcoholic fatty liver disease (NAFLD) [5].

Diagnosis of HFI is established with suggestive metabolic disturbances in urine analysis, blood tests, and clinical findings following dietary exposure to fructose, sucrose, or sorbitol. Specific tests include the fructose loading test, but it is rarely used as it increases the risk of severe hypoglycemia in patients. Liver biopsy is also possible; however, it is an invasive procedure. Genetic testing is recommended to confirm the diagnosis, which is highly effective and includes single gene sequencing, multi‐gene panels, and genome‐wide association studies [4, 6].

Management of HFI requires a multidisciplinary approach. The most important method in the management of HFI lies in the absolute avoidance of food containing fructose, sucrose, and sorbitol (FSS), whose efficacy is still unknown, as patients on an FSS‐free diet have developed fatty liver and growth failure even in clinically asymptomatic patients. It can also develop several nutritional deficiencies, especially vitamin C, which can be corrected by giving multivitamin supplements [6, 7].

This case report highlights the management of a toddler with HFI who presented with gastroenterology symptoms. The combined effort of the multidisciplinary team was necessary for comprehensive diagnosis and effective treatment. This case unveils the importance of prompt diagnosis and a collaborative approach in managing children with HFI with an aim to reduce the fatal complications and improve survival.

## 2 Case History/Examination

A 2‐year‐8‐month‐old female was brought to the pediatric gastroenterology department with a history of nausea, vomiting, progressive abdominal distension, excessive sleepiness, and decreased activity over a period of 2 to 3 weeks. Her symptoms were initially subtle, but feeding worsened, and parents noticed that the vomiting episodes constantly occurred after feeding, particularly after the consumption of sugary foods or drinks. This association led them to reduce such items in her diet, though the child's symptoms persisted, and her abdominal swelling became more prominent over time.

On physical examination, the child was found to be lethargic but hemodynamically stable. Her abdomen was distended, and the liver was palpable approximately 3 cm below the right costal margin, confirming hepatomegaly. There was no jaundice, ascites, or splenomegaly. Other systemic examinations were within normal limits, and there were no signs of neurological impairment or dehydration at the time of assessment.

## 3 Methods (Differential Diagnosis, Investigations, and Treatment)

Diagnostic workup was crucial in making the diagnosis and formulating a management plan. Laboratory investigations revealed a random blood glucose level of 50 mg/dL, indicative of hypoglycemia. Liver function tests showed elevated transaminases, with SGPT at 95 U/I and SGOT at 120 U/I, while alkaline phosphatase was also elevated at 516 U/I. A lipid panel showed hypertriglyceridemia with triglycerides at 395 mg/dL, a low high‐density lipoprotein (HDL) at 22 mg/dL, and low‐density lipoprotein (LDL) at 18 mg/dL. Total serum cholesterol was 92 mg/dL. Other investigations, including complete blood count, coagulation profile, serum ferritin, alpha‐fetoprotein, blood gas analysis, kidney function tests, electrolytes, and urinalysis, were all within normal ranges.

**FIGURE 1:**
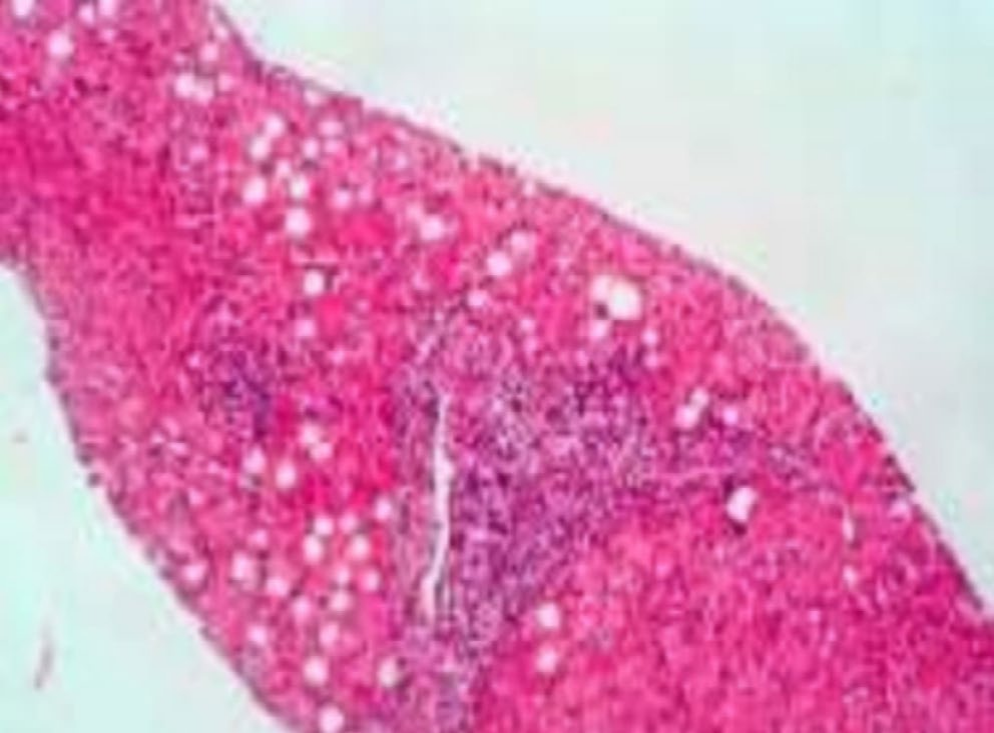
Histological examination revealed mild macrovesicular steatosis affecting 15%–20% of hepatocytes.

Abdominal ultrasonography showed hepatomegaly with increased echogenicity of the liver, suggestive of hepatic steatosis. Given the hepatomegaly, transaminase elevation, and lack of evidence for infection, autoimmune disease, or toxic exposure, a liver biopsy was performed for further evaluation. Histological examination revealed mild microvascular steatosis affecting approximately 15%–20% of hepatocytes (Figure 1). There was no mild to moderate chronic inflammation in both the portal and lobular areas. Early portal fibrosis (Stage 1–2 out of 4) was present, along with biliary ductular proliferation, indicating chronic liver injury likely due to a metabolic disorder.

The combination of clinical symptoms—particularly the strong correlation between symptoms and intake of sugary foods—along with biochemical evidence of hypoglycemia, hepatic steatosis, and histological changes, raised a strong suspicion of HFI. Although genetic testing for ALDOB mutations and aldolase B enzyme assays was not available at the time due to resource limitations, the clinical picture was sufficient to support a working diagnosis of HFI.

## 4 Treatment

Management involved immediate and complete dietary exclusion of fructose, sucrose, and sorbitol. The family received extensive nutritional counseling to help identify and eliminate offending substances from the child's diet. In addition to dietary modifications, the child was started on uncooked corn starch five times daily as a safe and slow‐releasing carbohydrate source to maintain stable blood glucose levels. Supplementation with vitamin D, vitamin E, and syrup ursodeoxycholic acid was also initiated to support liver function and prevent further hepatocellular damage.

## 5 Outcome and Follow‐Up

Over the following months, the child showed marked clinical improvement. Her energy levels improved, vomiting episodes ceased, and her abdominal distension gradually subsided. There were no further hypoglycemic episodes, and her appetite improved steadily. Growth parameters showed progressive improvement; she began to achieve developmental milestones that had been previously delayed. Liver function tests and lipid profiles normalized over time, and follow‐up abdominal ultrasonography showed significant reduction in liver size and improvement in hepatic echo texture.

At 1‐year follow‐up, the child remained symptom‐free, with normal physical activity and appropriate growth for her age. Her parents reported strict compliance with dietary restrictions, and routine monitoring continued with periodic assessments by the pediatric gastroenterology and nutritional teams.

## 6 Discussion

Hereditary fructose intolerance is often diagnosed in infancy or early childhood when fructose or sucrose‐containing foods are first introduced after breastfeeding [2]. Affected children present with nonspecific symptoms including vomiting, abdominal pain, lethargy, hypoglycemia, and failure to thrive, which occur in many diseases of early childhood leading to a significant challenge to early diagnosis [6]. Hepatomegaly and elevated liver enzymes are common findings and may raise suspicion for a metabolic liver disease, but histological findings such as macrovesicular fatty changes, inflammation, and early fibrosis support the diagnosis [8].

In our case, the diagnosis of HFI was based on a combination of clinical history, dietary triggers, laboratory findings, and liver histology. Although genetic testing is considered the gold standard with high sensitivity and specificity, especially to differentiate HFI from other metabolic conditions, many diagnoses in clinical practice are based on metabolic disturbances and clinical findings following dietary intervention [7, 9]. Importantly, the patient who is diagnosed early and adheres strictly to a fructose, sucrose, and sorbitol‐free diet may have a good prognosis and normal lifespan. Delayed diagnosis, however, can lead to growth retardation, progressive liver and renal disease, and may lead to death [10].

## 7 Conclusion

In summary, this case highlights the diagnostic and therapeutic challenges of managing pediatric HFI, especially in settings with limited access to genetic testing. It emphasizes the importance of clinical vigilance, continuous assessment, and multidisciplinary collaboration for early and accurate diagnosis. Despite advancements in diagnostics and therapy, dietary modification remains the cornerstone of management, serving both diagnostic and therapeutic purposes while promoting normal growth, developmental milestones, and long‐term adaptation. Strict adherence to dietary recommendations significantly improves outcomes, underscoring the value of personalized care and the need for further research to optimize treatment strategies and prognosis in this vulnerable population.

## Author Contributions

**Rajat Kumar Shah:** conceptualization, writing – original draft. **Sajjad Ahmed Khan:** conceptualization, writing – review and editing. **Bijita Aryal:** conceptualization, writing – review and editing. **Aakash Khatiwada:** writing – original draft. **Binita Gurubacharya:** writing – review and editing. **Shamin Parajuli:** writing – review and editing.

## Funding

The authors have nothing to report.

## Consent

Written informed consent was obtained from the patient to publish this report in accordance with the journal's patient consent policy.

## Data Availability

Data will be provided by the corresponding author upon reasonable request. Images are uploaded in the separate files.
